# Pediatric Airway Pathology

**DOI:** 10.3389/fped.2020.00246

**Published:** 2020-06-04

**Authors:** Shyan Vijayasekaran

**Affiliations:** ^1^Faculty of Medicine and health sciences, University of Western Australia, Perth, WA, Australia; ^2^Department of Otolaryngology Head and Neck Surgery, Perth Children's hospital, Nedlands, WA, Australia

**Keywords:** larynx, trachea, pediatrics, airway pathology, stridor, aspiration

## Abstract

Congenital or acquired disorders of the pediatric airway can affect the upper, lower, or entire airway. There are fundamental differences between the anatomy and physiology of the neonate, pediatric, and adult airways. Infants are not merely small adults in this respect and size, surface area, proportion, resistance, and compliance vary greatly between the age groups. A clear understanding of these significant differences and how they affect patients dependent on age is key to appropriate management.

## Introduction

There are fundamental differences between the anatomy and physiology of the neonate, pediatric, and adult airways. In the case of the pediatric airway, disorders may be both congenital or acquired, and the upper, lower, or entire airway may be affected. Airway obstructions can be relatively fixed or dynamic and may occur anywhere from the piriform aperture to the distal bronchi (Table 1). This review aims to highlight the key features of the neonate and pediatric airway and the significance of these differences with respect to airway disorders and obstruction.

**Table 1:** Disorders of the neonatal airway.

**Frequest diagnoses posing potential airway emergencies in the neonate**	
**Level of obstruction**	**Conditions**
Nasal	Piriform aperture stenosis Nasolacrimal duct cyst Choanal atresia Dermoid/encephalocoele Septal deformity Adenoidal hypertrophy
Pharyngeal	Tonsillar hypertrophy Vallecular cyst/lingual thyroid Vascular malformation Macroglossia Retrognathia/micrognathia Hypotonia
Laryngeal	Laryngomalacia Vocal cord paralysis Subglottic stenosis Laryngeal cleft Laryngeal web/atresia Ventricular cyst
Tracheal	Tracheomalacia +/– associated with oesophageal fistula Congenital tracheal stenosis +/– complete tracheal rings Vascular rings/slings
Craniofacial/multi-level obstruction	Trisomy 21 Bekwith Weidermann Crouzons/Alpert's syndrome Treacher Collins

*Adapted with permission from Lioy J, Sobol S, editors. Disorders of the neonatal airway: fundamentals for practice, p. 140.@ Springer, 2015*.

## Anatomy and Physiology

Size is an obvious key disparity between the neonate airway and that of the older child, or adult. To comprehend the impact of size difference it is important to have a clear understanding of the mechanism of flow in the neonate airway. Poiseuille's equation defines airflow through the airway where R is resistance, and r is the radius. When the airway radius is reduced, laminar airflow resistance is exponentially increased (to the fourth power).

$$\begin{array}{l} \text{R }=\;1/{\text{r}}^{4} \end{array}$$

Turbulent flow further exacerbates this effect and, with turbulence, resistance is increased to the fifth power as the radius is reduced. For example, airflow resistance in an infant with 1 mm of circumferential edema, will be increased 16-fold, while in the presence of turbulent flow, resistance, and consequently work of breathing, increases 32-fold. Thus, the effect of a minor airway narrowing in the neonate as a result of a bronchoconstriction or respiratory infection has a disproportional impact on airflow resistance.

Neonates also have a distinct physiology relative to the older child and adult and these differences can exacerbate the effects of airway disorders. Respiratory function in the neonate is influenced by differences in chest wall compliance, functional residual capacity, and muscle fiber type. Healthy term neonates have a lower functional lung capacity due to the mechanical disadvantage of a more compliant rib cage (1, 2). In addition, the diaphragms of the neonate are elevated compared with the older child due to the larger size of their abdominal contents and reduced lung volumes (1, 2). The respiratory reserve of the neonate is also reduced when compared to the older child or adult due to significantly smaller lungs relative to their increased metabolic needs and oxygen consumption (3).

Infants are described as obligate nasal breathers due to their low-lying posterior soft palate and enlarged epiglottis that arises from the close proximity of the hypopharynx and nasopharynx. It is this normal anatomical relationship that reduces the risk of aspiration. During the first year of life, growth leads to an increase in the space between the hypopharynx and nasopharynx. Although described as obligate nasal breathers, preferential nasal breathers may be a more apt description, as recent studies describe the ability of infants to breathe through the mouth during both spontaneous breathing and nasal occlusion (4, 5).

## Nasal Obstruction

Neonatal nasal passage obstruction may be the result of a number of congenital abnormalities. For example, choanal stenosis/atresia, which is a bony obstruction of the posterior nasopharynx, occurs in approximately one in every 5000–7000 births. The neonatal patient typically presents with respiratory distress and cyanosis when mouth breathing, which improves with crying. An inability to pass a catheter through the nasopharynx supports the diagnosis, which can be confirmed by computed tomography (CT) (6–8). A prenatal diagnosis has recently been found to be possible in some instances with the use of a high-definition prenatal ultrasound. Once a diagnosis has been made, it is imperative that a full genetic evaluation is carried out as choanal atresia has been associated with a number of congenital syndromes, including CHARGE (Coloboma of the eye, Heart defects, Atresia of the choanae, Restricted growth, Genital anomalies, Ear anomalies). In the case of bilateral choanal atresia surgical intervention can be implemented in the perinatal period (9, 10), the outcome of which depends largely upon the degree of bony atresia, the size of the neonate, and the frequency of restenosis (11).

Another congenital abnormality is pyriform aperture stenosis, which is characterized by narrowing at the anterior nasal bony inlet (8, 12, 13), and often found in association with other midline defects, including holoprosencephaly, hypopituitarism, and septo-optic dysplasia (14, 15). The need for surgical intervention can be predicted with an 88% sensitivity and specificity if the neonatal pyriform aperture width is <5.7 mm (16).

Masses in the nasal passages also give rise to nasal obstruction and include nasolacrimal duct cysts, nasal encephaloceles, and nasopharyngeal tumors, namely teratomas (17, 18).

## Oropharynx

The neonate oropharynx comprises the tongue and palatal structures. Airway obstructions do not typically originate from the palate itself, but tumors that emanate from the palate may, in rare instances, result in an oropharyngeal obstruction. The neonatal tongue is proportionately larger and potentially more obstructive than that in the adult. As a result, airway obstructions in the neonate are far more often associated with the tongue (3). In addition, neonatal genetic syndromes, including trisomy 21 and Beckwith–Weidemann syndrome (BWS), are often associated with macroglossia. In the case of BWS, the macroglossia may become worse over time and, in some instances, may necessitate the need for a nasopharyngeal tube, tongue volume reduction, midline posterior glossectomy or a tracheostomy (19).

Airway obstruction in the neonate may also occur in cases of micrognathia or retrognathia. These deformations give rise to a recessed mandible and may displace the tongue to the rear of the oropharynx, resulting in obstruction. Both retrognathia and micrognathia are often found in conjunction with genetic craniofacial syndromes, such as Pierre Robin sequence or Stickler's syndrome, in which the micrognathia is accompanied by a cleft palate (20). The extent to which the neonatal tongue can contribute to airway obstruction is often under-appreciated. Thus, to assess the severity of the obstruction and guide treatment, it may be useful to conduct formal sleep studies. Obstructions have traditionally been relieved by tongue–lip adhesion, or bypassed by a tracheostomy. However, promising results have recently been achieved with mandibular distraction osteogenesis, which may present a potential alternative therapeutic option (21). In micrognathia cases where endotracheal intubation is difficult to perform, the obstruction can be relieved in the short term by prone positioning or placement of an oral or nasopharyngeal airway.

## Supraglottis

The supraglottis comprises the vallecula, the epiglottis, the arytenoid cartilages, and the aryepiglottic folds.

The difference between the neonatal epiglottis and that of an adult is that it is proportionally longer, narrower, larger, less flexible, and often omega-shaped. In the condition known as laryngomalacia (LM), a combination of an omega-shaped epiglottis, short aryepiglottic folds, and/or redundant supra-arytenoid tissue and cuneiform cartilages, causes a collapse of the supraglottic structures into the glottis during inspiration (1).

Laryngomalacia is the most common cause of stridor (60%) among the pediatric population (22). Patients characteristically have a high pitched intermittent inspiratory stridor, which is exacerbated by agitation, crying, the supine position, and feeding. In ~5–20% of cases, respiratory distress, hypoxemia, or obstructive sleep apnea may occur (23). Feeding difficulties, including coughing, choking, regurgitation and failure to thrive can occur due to problems with suck-swallow-breathe coordination. Diagnosis is made by awake bedside flexible nasopharyngoscopy, showing inspiratory collapse of the supraglottic structures. This collapse is governed by the physics of flow through a tube, i.e., the Bernoulli principle (reduced intraluminal pressure secondary to increased flow velocity) and the Venturi effect (reduced intraluminal pressure due to a constriction to flow). LM may be exacerbated by crying, feeding, and the supine position. LM may also aggravate gastroesophageal reflux disease, resulting in failure to thrive or obstructive sleep apnea (23). A supraglottoplasty performed early in neonates with severe laryngomalacia, with release of tight aryepiglottic folds, or early removal of redundant tissue, often alleviates the airway obstruction (24).

**Figure 1:**
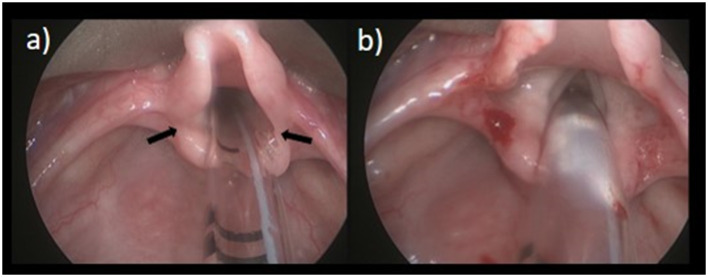
Laryngomalacia. **(a)** Pre OP; **(b)** Post division of aryepiglottic folds.

A supraglottoplasty aims to reduce the redundant or obstructive supraglottic tissue, therefore reducing inspiratory collapse of the airway. This is done under general anesthesia, with any one, or combination, of the following according to the anatomical findings and the area of airway obstruction: removal of redundant arytenoid tissue, division of aryepiglottic folds, or epyglottopexy (Figure 1). Surgical methods vary and can include cold instruments, a microdebrider, and CO_2_ laser. The majority of patients obtain relief of obstructive airway symptoms after surgery with resumption of a normal diet for age (25). Reflux treatment is recommended for 4–6 weeks post-operatively. Children with severe LM, or those with comorbid neurological or syndromic conditions, may have persistent obstruction. In these instances a revision supraglottoplasty is occasionally required and is preferable to over-resection of tissue that may result in prolonged aspiration or feeding disorders. Surgical complications are rare, with supraglottic stenosis being the most concerning, at a rate of up to 3.9% of cases (26). A tracheostomy may be required in cases of severe LM that are not alleviated following supraglottoplasty. Feeding intervention including thickened formula or breast milk, feeding in an upright position, and speech language pathology (SLP) evaluation, may be warranted.

Bronchoscopy is indicated to rule out associated airway abnormalities (12–64%) (27), most commonly subglottic stenosis, vocal cord palsy, or tracheomalacia. This condition is usually self-limiting, with an improvement of symptoms as the infant airway grows.

## Glottis and Sub-Glottis

In all cases the larynx is the entrance to the lower airway but in the neonate and infant it is positioned higher in the neck relative to the cervical spine, compared with the adult larynx (1, 2). A further difference is that the neonate and infant larynx is proportionally smaller to that of the older child, while the surrounding arytenoids and aryepiglottic folds are relatively larger (2, 8). As the infant grows into a child the larynx descends, and by the age of six it is similar to the that of an adult. A typical larynx is cone- or funnel-shaped and attached to the trachea by the cricoid cartilage. In a young infant the cricoid cartilage is typically the narrowest point in the airway, while the glottic opening is the narrowest point in adults. The cricoid is a complete ring of cartilage (~2 cm in length) that surrounds the trachea and starts below the vocal cords. It is at the level of the cricoid that resistance to the endotracheal tube during tracheal intubation of the neonate is encountered and this is what ultimately determines the suitable size of the endotracheal tube. Uncuffed endotracheal tubes are normally used in neonates due to this cricoid narrowing, although some centers use cuffed tubes, but always with strict pressure monitoring.

### Vocal Fold Paralysis

Vocal cord paralysis (VCP) is the second most common laryngeal abnormality. With *unilateral* VCP, patients present with a weak cry, stridor, aspiration, dysphagia, and feeding difficulties. *Bilateral* VCP is typically more severe in presentation with increased obstruction possibly requiring intubation. This can result from birth trauma, cardiothoracic procedures, neurological disease, or from idiopathic etiology. Iatrogenic left VCP is well-documented after cardiac surgery, specifically patent ductus arteriosus ligation or repair. In every child with a bilateral VCP, intracranial or intrathoracic pathology needs to be excluded, thus magnetic resonance imaging of the brain down to the mediastinum is recommended. A flexible nasendoscopy in the awake patient showing unilateral or bilateral vocal cord immobility is diagnostic. Around 50% of children with bilateral VCP will need a tracheotomy to secure the airway and 48–62% of children will have spontaneous recovery of vocal cord function over time (28). Delayed vagal nuclei maturation has been proposed as the likely mechanism to explain the vocal cord recovery. If a tracheotomy had been performed these patients are able to be decannulated (29).

Patients with unilateral VCP usually compensate for the purpose of feeding and voice with no intervention. They are generally followed closely by the otolaryngology team in conjunction with SLP for feeding management. Children who fail positional feeding or thickened feeds may require nasogastric feeding. Since recovery of vocal cord movement is expected in up to 62% of cases within 24 months, postponing invasive permanent procedures is usually warranted. The greatest challenge is selecting a surgical approach that promotes a safe airway, and preserves voice and swallowing function.

There are many procedural options for *bilateral* VCP involving both endoscopic and open procedures to the vocal cords, arytenoids or cricoid to resect or augment portions of the larynx as needed. These include vocal fold botulinum toxin injection, suture lateralization, vocal cordotomy, arytenoidectomy, and cricoid split with, or without, cartilage augmentation. Apart from posterior cricoid split and costal cartilage graft augmentation, most procedures are considered destructive to the vocal fold architecture. There has been a trend to early reconstruction to avoid a tracheostomy in selected patients in whom a posterior cricoid split with costal cartilage grafting may be utilized with good outcomes (28, 29).

In the case of *unilateral* VCP, the mainstay of surgery involves augmenting the vocal fold with fillers (such as hyaluronic acid), or surgically medializing the vocal folds. Alternatively, re-innervation procedures may be considered. Reinnervation is more successful in unilateral VCP than in bilateral VCP (30).

### Subglottic Stenosis

Subglottic stenosis (SGS) is an airway narrowing at the level of the cricoid cartilage, 4 mm (requiring a 3.5 mm endotracheal tube) or less in full term babies, and 3 mm or less in premature infants (31). It is the third most common airway abnormality of the larynx, following laryngomalacia and vocal cord paralysis, and the most common laryngeal anomaly requiring tracheotomy in children under 12 months. SGS is classified as congenital or acquired. Congenital SGS results from cricoid cartilage malformation without previous endotracheal intubation. Acquired SGS (90%) is generally secondary to endotracheal intubation (32); a complication of successful neonatal resuscitation with prolonged intubation. Risk factors for SGS include a higher number of intubations, a longer duration of ventilation, unplanned extubation, traumatic intubation, and oversized endotracheal tubes (33).

**Figure 2:**
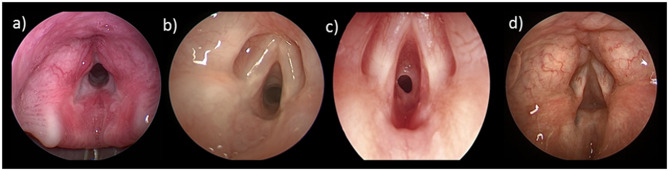
Cotton-Meyer Grading of Subglottic Stenosis: **(a)** Grade *I* < 50% obstruction; **(b)** Grade II 51–70% obstruction; **(c)** Grade III 71–90% obstruction; **(d)** Grade IV 100% obstruction.

Subglottic stenosis presents with biphasic stridor, respiratory distress, and/or failure to extubate. A history of previous intubations, difficult intubation, and recurrent croup-like symptoms are red flags for acquired SGS. Direct visualization with microlaryngoscopy, bronchoscopy under general anesthesia, and airway sizing with age appropriate endotracheal tubes is required to diagnose SGS and plan treatment. The severity of the disease can be classified according to the Cotton-Meyer grading system (Figure 2).

Once a diagnosis is made, further management and surgical treatment varies widely depending on severity of the disease. Infants with mild symptoms and Grade I or II SGS may be followed conservatively. More severe disease and symptoms may require surgical intervention to widen the airway. A tracheotomy may be required to secure the airway and permit the SGS to mature prior to further surgical intervention.

Surgical management may be endoscopic, open, or a combination of both, according to the degree of stenosis. In cases of acquired SGS endoscopic balloon laryngoplasty is considered less invasive, easier to perform, and simpler to manage post-operatively compared to open approaches. Under general anesthesia a balloon catheter is introduced through the subglottic narrowing and insufflated to obtain a mechanical dilatation of the subglottis. Endoscopic dilation may be effective for patients with mild severity and a short stenotic segment (10). This technique can be assisted by endoscopic cricoid splits (anterior or anterior and posterior) with intralesional corticosteroid injection and has been associated with high rates of decannulation and less morbidity than open approaches. It often has to be performed repeatedly to obtain long term patency (31, 32).

Grade II and IV SGS may require open laryngotracheal reconstruction (LTR). In this procedure autologous cartilage is used to augment the larynx and proximal trachea. Different types of cartilage grafts have been described (costal, thyroid alae, auricular cartilage, and hyoid bone) with costal cartilage used most commonly. The operation consists of augmenting the laryngotracheal framework with an anterior and/or posterior midline incision of the cricoid cartilage and insertion of the cartilage graft to expand the airway. The procedure may be single stage, i.e., widening the SGS without a tracheostomy or with simultaneous removal of the tracheostomy, or double staged. Several factors affect the choice of single or double stage. Patient with multi-level airway stenosis, comorbidities, potentially difficult reintubation, and surgeon preference are indicators for double stage surgery (34). For single stage procedures, patients are kept intubated in ICU for 4–7 days with a planned laryngobronchoscopy before or after extubation. Double-stage LTR is similar, however a tracheostomy is performed and/or maintained until the airway has healed and is safe and patent for decannulation.

If needed, endoscopic balloon laryngoplasty and other ancillary techniques can be used to facilitate decannulation. LTR is often the best treatment for SGS, with high success and decannulation rates up to 80%, even for severe SGS. Partial cricotracheal resection (pCTR) is a surgical option for patients with severe grade III and IV SGS, and those who have failed a previous LTR. In this procedure, the stenotic segment (anterior cricoid arch and tracheal rings) are resected and the proximal and distal segments are re-anastomosed. A double stage CTR is preferred when there is involvement of the glottis and associated systemic comorbidities and delayed decannulation is preferable. An extended CTR is utilized in cases where the subglottic and glottic airway (posterior glottic stenosis or fixed crico-arytenoid joints) is compromised. In this procedure costal cartilage is used to augment the posterior glottis in addition to the cricotracheal resection.

### Laryngeal Webs and Atresia

Laryngeal or glottic webs are rare (~5%) congenital anomalies resulting from incomplete recanalization of the primitive laryngeal lumen at 8 weeks gestation. There is a spectrum of anomalies, determined by the amount of recanalization that occurred before the embryological process was interrupted (31). This ranges from a small anterior web to a complete laryngeal atresia. The most common site of involvement is the anterior glottis, often extending inferiorly. Symptoms range from mild dysphonia and stridor to airway compromise. Total obstruction is known as congenital high airway obstruction syndrome (CHAOS), characterized by enlarged echogenic lungs, inverted diaphragms, massive ascites and fluid filled airways. It can be diagnosed prenatally with a fetal MRI. Delivery is by *ex-utero* intrapartum procedure (EXIT) with an immediate tracheotomy to secure the airway while the neonate remains on placental support.

**Figure 3:**
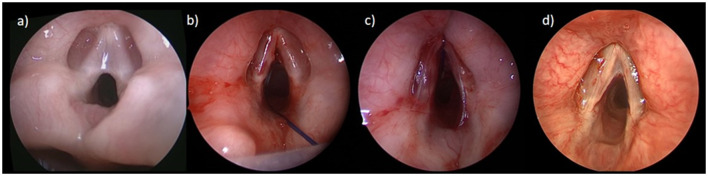
Laryngeal web: **(a)** anterior laryngeal web; **(b)** intraoperatively after division of the web; **(c)** with silastic keel in place; **(d)** 1-year postoperative with a small asymptomatic residual web.

Laryngeal webs are diagnosed on microlaryngoscopy. They are typically anterior and partially obstructing the lumen. Incidental webs may be detected during laryngoscopy or intubation. There is an association between anterior glottic webs and 22q.11.2 deletion syndrome, therefore karyotyping for microdeletion of chromosome 22q.11 is warranted (35). Glottic webs are classified by the Cohen Classification (Table 1), taking into account the degree of obstruction and extension into the subglottis. Treatment depends on symptom severity and exam findings with the primary objective to secure the airway and preserve vocal quality. Those with Type 1 webs may be observed clinically, whereas those with more significant webs generally require surgical intervention with either endoscopic or open procedures. Many techniques are described to divide the web and a keel, a piece of silastic or silicone material may be placed into the anterior commissure between the edges to prevent scarring and recurrence, the most common complication in webs (Figure 3). In thicker webs with a subglottic extension, an open approach with laryngofissure and laryngotracheal reconstruction with anterior cartilage graft and stenting is indicated. A tracheotomy may be performed to allow laryngeal growth before definitive reconstruction.

### Laryngotracheo-Esophageal Clefts

A laryngo-tracheo-esophageal cleft (LC) is a congenital malformation characterized by an abnormal, posterior, sagittal communication between the larynx and the pharynx, possibly extending inferiorly between the trachea and the esophagus. The estimated annual incidence of LC is 1/10,000 to 1/20,000 live births, accounting for 0.2 to 1.5% of congenital malformations of the larynx. Five types of laryngo-tracheo-esophageal cleft have been described based on the inferior extension of the cleft, which typically correlates with the severity of symptoms.

**Figure 4:**
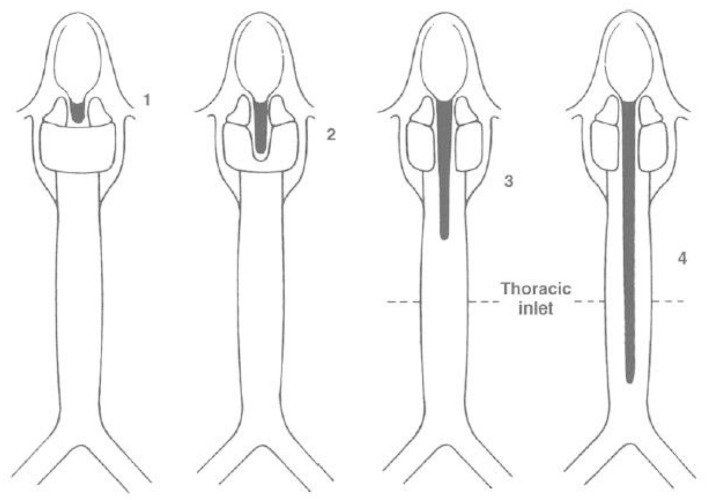
Benjamin—Inglis Classification. Sandu Modification of the original Benjamin-Inglis Classification. Type 0: submucosal cleft. Type I: supraglottic, interarytenoid cleft, above the vocal fold level. Type II: cleft extending below the vocal folds into the cricoid cartilage. Type III a: cleft extending through the cricoid cartilage but not into the trachea. Type III b: cleft extending through the cricoid cartilage and into the cervical trachea. Type IV: cleft extending into the thoracic trachea, potentially down to the carina.

Depending on the severity of the malformation, patients may present with aspiration and feeding issues for shorter clefts (Type 0 and I), to marked dysphagia, stridor, dysphonia, dyspnea and cyanosis for longer clefts (II-III), to life threatening distress requiring intervention within the first few hours of life (type IV) (Figure 4).

Associated syndromes include Optiz G/BBB, 22q11 deletion, Pallister-Hall, VACTERL association, and CHARGE syndrome. The most common associated anomalies include esophageal atresia, trachea-esophageal fistula, and tracheomalacia. Diagnosis is made on laryngoscopy and bronchoscopy, and aided by contrast radiology and an awake nasendoscopy, with or without endoscopic evaluation of swallow.

Thickened fluids, GORD treatment and the use of the postprandial upright position have been successful for the treatment of mildly symptomatic type I cleft children. Patients with a symptomatic type I or II cleft are usually treated as above with the addition of nasogastric tube feeding, but will almost always also require either endoscopic, or open, surgical intervention. Endoscopic treatment may be palliative with an injection of hyaluronic acid filler or a formal surgical repair using suspension microlaryngoscopy without an endotracheal tube in a patient breathing spontaneously. Parenteral nutrition may temporarily be required in some cases of significant type III or type IV clefts where the risk of aspiration is extremely high. High grade clefts often require a mid- to long-term gastrostomy (often with fundoplication) and formal open surgical repair. Frequently a tracheostomy is needed to manage tracheomalacia. Gastric division with a proximal drainage tube and distal gastrostomy have also been proposed for type IV LCs. The anterior trans-laryngotracheal approach, raising asymmetric mucosal flaps, with excision of excess mucosa and non-overlapping suture lines, with or without interposition grafts, is favored as it avoids the risk of recurrent laryngeal nerve injury (36).

## Author Contributions

SV was involved in the concept and drafting of the entire manuscript.

## Conflict of Interest

The author declares that the research was conducted in the absence of any commercial or financial relationships that could be construed as a potential conflict of interest.
